# Enhanced Surgeons: Understanding the Design of Augmented Reality Instructions for Keyhole Surgery

**DOI:** 10.1109/VRW58643.2023.00031

**Published:** 2023-03-25

**Authors:** Christoph Davis, Soojeong Yoo, Athena Reissis, Matthew J. Clarkson, Stephen Thompson

**Affiliations:** Wellcome / EPSRC Centre for Interventional and Surgical Sciences, University College London (UCL), United Kingdom

**Keywords:** Human-centered computing, Visualization, Visualization techniques, Treemaps, Human-centered computing, Visualization, Visualization design and evaluation methods

## Abstract

It is important to understand how to design AR content for surgical contexts to mitigate the risk of distracting the surgeons. In this work, we test information overlays for AR guidance during keyhole surgery. We performed a preliminary evaluation of a prototype, focusing on the effects of colour, opacity, and information representation. Our work contributes insights into the design of AR guidance in surgery settings and a foundation for future research on visualisation design for surgical AR.

## 1 Introduction

Minimally invasive (or keyhole) surgery allows for surgeries to be performed by making only small incisions. For abdominal surgery a rigid endoscope (known as a laparoscope) is used to visualise the patient’s internal anatomy and surgical instruments. The smaller incisions used allow a faster recovery time in comparison with open surgery. The adoption of keyhole surgery, however, reduces the amount of information available to the surgeon during surgery, due to the reduced field of view of the cameras used and the inability to directly touch the anatomy. The use of image guidance for keyhole surgery aims to overcome these limitations, making keyhole surgery more effective. This paper is principally concerned with enhancing keyhole surgery using augmented reality derived from pre-operative scans (CT and MRI).

AR enables a composite image to be displayed on one screen through the combination of a computer-generated image and a live real world image [5], and has found extensive applications within the medical field [8] [12]. AR for laparoscopic surgery overlays the internal anatomy of an organ onto the real time image captured by the laparoscope [17, 19]. Research into the use of AR in surgery has grown significantly in recent years and improvements in the technology have the potential to improve patient outcomes.

The majority of augmented reality systems for keyhole surgery propose projecting surface renderings of the internal anatomy onto the live video feed [1]. A key technical challenge to enable this is registration, where the geometric relationship between the surface models and the visible anatomy is estimated, [9]. Much of the literature on AR for surgery is focused on the technical difficulties of the registration, rather than more holistic consideration of system performance when used by a surgeon. Visualisation design remains a neglected element of research into AR for keyhole surgery.

Because the focus is usually on the technical difficulties of registration the majority of proposed surgical AR systems use fairly simplistic rendering, i.e. solid models and wireframes with various levels of opacity and various colours [19] [10]. This paper focuses on these visualisation methods with the aim of creating some baseline results which can be compared to more sophisticated visualisation methods in future work. This work is needed to understand the human factors surrounding the design of AR content for keyhole surgery settings. This is a crucial aspect to understand as poorly designed systems could potentially distract surgeons from their task which can lead to complications in the surgical procedure.

## 2 Related Work

The majority of research on AR for keyhole surgery focuses on the engineering challenge of achieving an accurate and reliable overlay of the AR content onto the operation scene. Whilst some authors [6,20,29] have provided explicit consideration to visualisation design in their works, the insights were primarily focused on quantitative performance of their respective systems. However little work to-date has focused on gaining understanding of the user experience these systems can provide. Work by Fischer et al. [10] provides a useful study of how visualisation design can affect the task of manually aligning the augmented reality model to the visible anatomy. Birlo et al. [2] provide an extensive review of some human factors present in AR for surgery, however, it is difficult to go from there to more specific conclusions on visualisation design for keyhole surgery. For example, several authors [13–15,24] have identified depth perception as a significant challenge in augmented reality for surgery, however we still lack the specific methods to address this in surgical AR.

There is a significant body of work on the use of AR in both open and keyhole surgery. There are also several well designed studies looking at the impact of AR on certain clinical outcomes. These studies tell us little, however, about the best way to design AR visualisation to maximise the effectiveness of a given AR system. There are problems with generalising some studies plus the more specific problem of recruiting sufficient volunteers to study multiple design options when the tasks used require a high baseline skill level. Our aim is to create a more general methodology together with the tools (hardware, software, and data) that can be applied to multiple studies in AR guidance for surgery.

The original focus of this study was the occurrence of inattentional blindness (IAB) during surgery. IAB describes the situation in which important details are missed due to the user’s attention being held elsewhere, [16, 25]. IAB has been demonstrated to have significant negative impacts during common activities such as driving a vehicle [18] and there is a growing body of work examining the impact of IAB in surgery, both with and without AR. Dixon et al. [7] performed a two armed study where volunteers were asked to perform an endoscopic surgical task on a cadaver, one arm of the study performed the task with AR guidance, one arm without. Dixon et al. showed that whilst AR helped achieve faster surgery, it also led to an increase in the number of participants who missed critical information in the surgical scene itself, in this case unusual anatomy of the optic nerve and a misplaced surgical screw. Dixon et al. only used one visualisation design for the AR and did not attempt to examine whether there may be visualisation designs that would both aid faster surgery and reduce the incidence of IAB. Pandit et al. [21] recently demonstrated the occurrence of IAB during neurosurgery with no AR guidance, but more interestingly that this could be reduced with mindfulness training. The results from Pandit et al. send a clear message that when studying IAB it is critical to control for the skills and mental state of the participants. We have attempted to do this with our study design. A key factor in understanding IAB is that the occurrence of IAB is related both to the strength of the distracting stimulus [7, 18] and the users’ base mental load.

One difficulty with the studies described by Dixon et al. and Pandit et al. [7, 21] is that the studies require the recruitment of skilled surgeons. This makes it difficult to extend their studies to larger experiments designed to better understand what specific factor contributed to their results and how system design can be improved. A key contribution of our work is the ability to use more abstract representations of surgical tasks. Our aim is to design these abstract tasks to induce similar cognitive loads to those experienced by surgeons performing AR guided surgery.

### 2.1 Summary and Research Gap

In this research, we explore how to utilise AR information overlay for guiding users through laparoscopic surgery tasks. We developed and evaluated an AR prototype which overlays points of interest over a phantom liver within a phantom patient. We performed a user study of the prototype where we were gaining participant feedback about the experience, particularly around the colour, opacity, and representation of the AR overlay. We also recorded task completion time to see if this was effected by overlay design, along with the occurrence of IAB. This work presents the first step towards better understanding how virtual information should be represented in AR systems for keyhole surgery. Our key contribution is design considerations of AR overlays for laparoscopic surgery, which can help optimise the design of future AR systems for this purpose.

## 3 Design and Development of AR Keyhole Surgery Instruction

**Figure 1:**
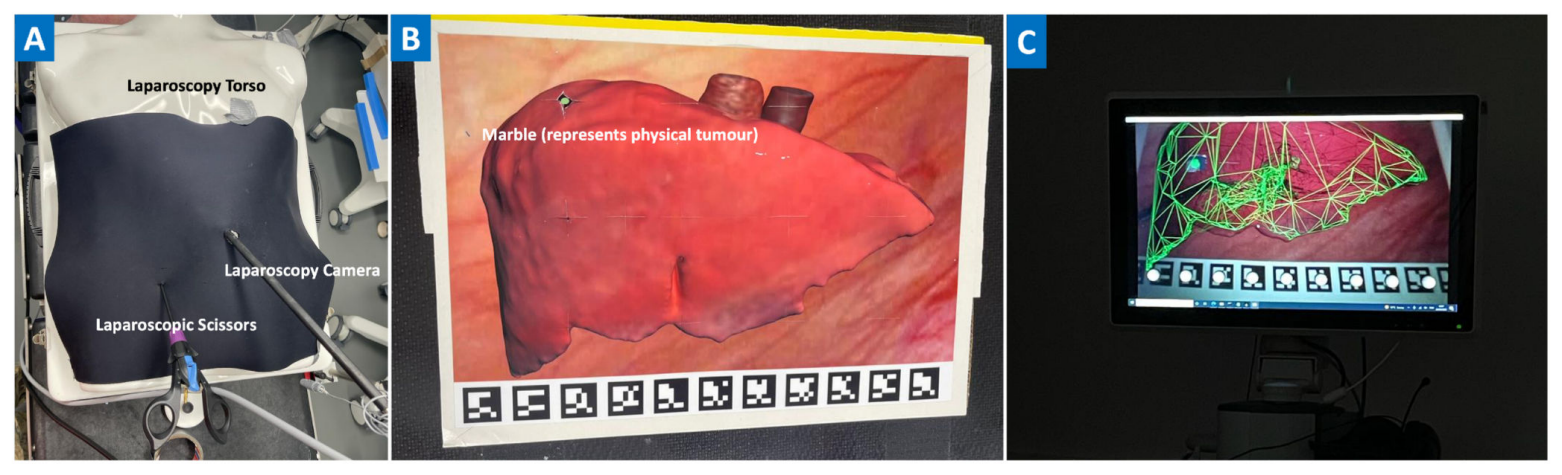
**A:** Study setup, a laparoscopy plastic torso with laparoscopy camera and scissors to simulate keyhole surgery. **B:** View inside the plastic torso with no augmented reality, showing the cardboard phantom with a marble in the cavity representing the tumour. During the experiment this view is covered by a neoprene sheet to represent the abdominal wall, so the participants can only see what is happening via the video screen. **C:** An example of one of the augmented reality overlays used, here a green wireframe to represent the liver, and a solid, low opacity, green tumour.

The study was setup using a laparoscopic torso simulator^1^ to mimic the setting of keyhole surgery (Figure 1A). Inside the plastic torso we placed a phantom to represent the liver with a tumour. Participants were asked to grasp and remove a simulated tumour (simulated with a marble) from the cut out area, using AR guidance. The AR functionality was enabled through a laparoscope camera to track the 2D liver model. The laparoscope camera was held static during the experiment to try and limit the task load on the participants. During laparoscopic surgery the surgeon does not usually move the laparoscope, as this task is done by a more junior surgeon under verbal instruction from the senior surgeon.

The liver model consisted of a 2D print on photographic paper attached to a shallow cardboard box which provides a recess in which a marble was placed. Several images for the phantom design were reviewed before settling on the image shown in Figure 1B which was created by texture mapping a solid model derived from a CT scan from a human patient. We preferred this slightly abstract view to images taken directly from laparoscopic video as it allowed easier interpretation for our non expert volunteer group. The AR overlay utilised the solid model of the same liver along with a tumour derived from the original CT data but scaled and moved to suit our purpose. The use of a flat liver phantom rather than a 3D model reduces the realism of the task, however given that the entire task is performed using a 2D monitor we are confident that the results will be transferable to more complex models that could be used in future work.

We used SciKit-SurgeryBARD [26] to project an augmented reality overlay onto the video monitor, as shown in Figure 1C. Three colours of overlay were used, pure red, pure green, and pure blue. Two levels of overlay opacity were trialled (low: 30% and high: 100%). Two model representations were used, wireframe and surface model, due to their prevalence in the surgical AR literature. As these methods obscure the underlying anatomy they are unlikely to be the ideal visualisation method, however, they are representative of the state of the art in this field.

SciKit-SurgeryBARD is open-source software, part of the SciKit-Surgery [28] project. SciKit-Surgery follows sustainable software best practice [22] to provide reusable software libraries for research, education, and translation for applications in image guided surgery. SciKit-SurgeryBARD combines object tracking using ArUco [11] markers and OpenCV [3] with augmented reality visualisation using VTK [23]. As the tracking and visualisation are performed using the same camera, SciKit-SurgeryBARD is capable of sub millimetre overlay accuracy, making it suitable for many tasks in augmented reality research and education. SciKit-SurgeryBARD allows visualisation parameters (object opacity, representation, and colour) to be specified in a JSON [4] configuration file, enabling consistent and reproducible results. The configuration files and phantom design described in this paper are archived and publicly available [27], enabling other researchers to easily replicate our study.

We aimed to gain insights into the benefits of AR in keyhole surgery, therefore, our implementation and study setup ensured that we simulate similar interface and interaction for keyhole surgery.

## 4 Study Design

We designed a within-subject user study, where each participant trialled our prototype across three different AR overlay conditions, by varying overlay type (solid and wireframe), colour, and opacity. In order to reduce the effects of learnability, we randomised the colour (red, green and blue) and opacity (low or high) of the overlay for each condition. There was no time limit and participants could stop at any time and move on to the next condition.

The order was varied across subjects to give balanced order of use, to account for potential order effects. For each condition, participants were asked to grasp and remove a simulated tumour from the cut out area, under AR guidance. While participants were using each condition, we recorded the total time spent in each condition type. We collected qualitative feedback through interviews, at the very end of all the conditions, to seek participants’ perception of the AR tumour overlay for keyhole surgery.

**Figure 2:**
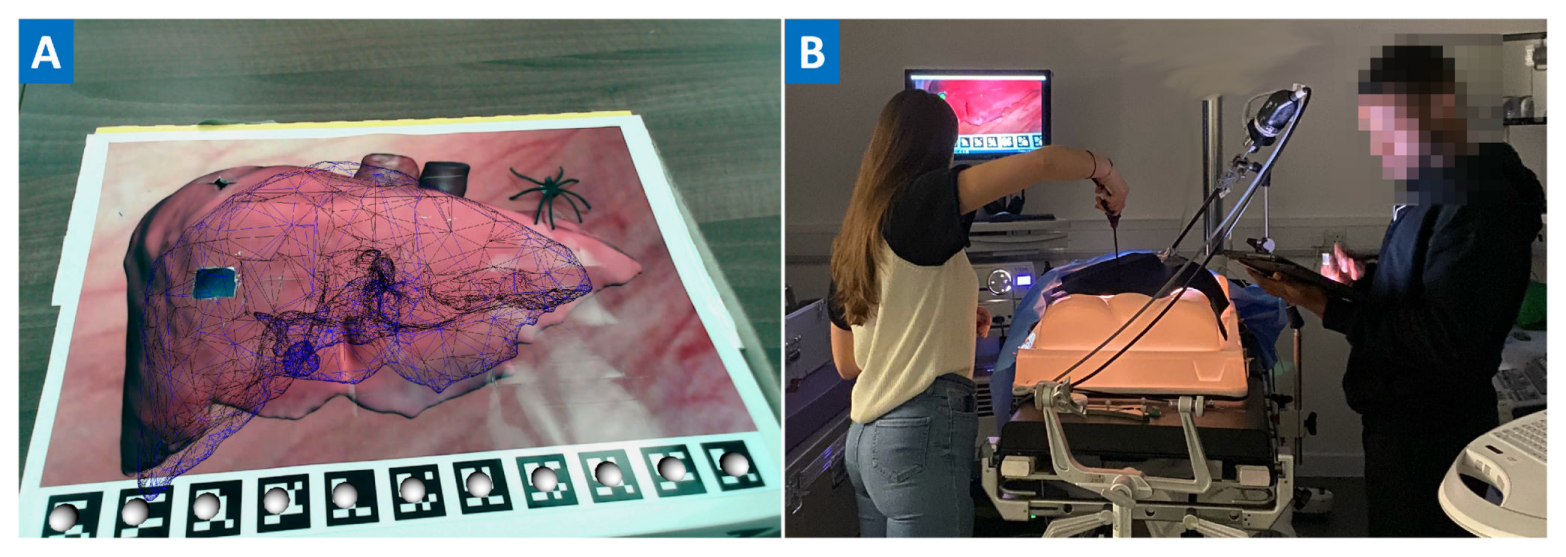
**A:** An example overlay using a blue wireframe model of the liver with a black plastic spider placed on the scene. The spider was used to test for inattentional blindness. **B:** An image of the experiment in progress (pixelation to maintain author anonymity). The user, at left, is attempting to perform a simulated surgical task on the white anatomical phantom to their right. The user is using a rigid laparoscopic tool, which consists of a pair of pincers actuated by the handle of the tool. The user cannot see directly into the phantom, but is instead using video images fed from the laparoscopic camera on their right and shown on the screen in front of them.

To test for the occurrence of IAB we also placed a black plastic spider withing the surgical scene, see Figure 2A. At the end of the study the participants were interviewed to gather qualitative data, which included being asked whether they had observed the spider. The spider was chosen during the study design process. Because we wanted to recruit non-expert users, more realistic objects like surgical clips were not always identified as foreign objects. The spider was chosen as we believed it to sufficiently out of place to be noticed by non-expert users.

We recruited 12 participants who were given a task designed to simulate laparoscopic surgery with AR guidance. The study was completed under ethical approval (Z6364106/Computer Science/2019/04/19) and all participants gave informed consent. Participants were asked about any visual impairments, but not specifically about colour blindness, which may be relevant given the contrast between the foreground and background images. Most of the volunteers were students studying Biomedical Engineering, so had some understanding of anatomy and interventional healthcare, but no surgical training. We created a simulated task that would represent a surgical task but at a level of abstraction relevant to the volunteers used.

Figure 2B shows an example of our experimental setup. Key points to note are that there is a significant cognitive load caused by the misalignment of the motor and visual axis [30], and the requirement to use a rigid pivoted tool to perform all actions. Experienced laparoscopic surgeons become accustomed to this, however our volunteers were not accustomed to it.

## 5 Results and Discussion

### 5.1 Task Completion Time

Each participant performed the task 3 times, giving a total of 36 data points. Mean task completion time was 42 seconds with a standard deviation of 41 seconds, maximum of 188s, minimum of 9s. We observed a statistically significant (p = 0.011) increase in task completion time for first task completed by each participant (mean = 66 seconds) indicating a strong learning effect. Future studies should include a pre-trial training session. Our results indicate that it would be sufficient to include a single pre-trial training run into future studies.

Our results do not demonstrate any statistically significant correlations between task completion time visualisation method or visualisation colour. Including outliers the red display gave a faster completion time on average which supports the qualitative results on the selection of overlay colour, however significantly more data would be required to demonstrate this.

### 5.2 Inattentional Blindness

After completing 3 tasks each participant was asked if they noticed anything out of place in the experiment. Only one participant noticed the spider. This surprised us, and demonstrates that IAB is a real problem that can be replicated in these studies. Given the small sample size it is not possible to extrapolate further at this point. Ideally we could design future studies with a finer balance between object prominence and task load to get a more useful result in terms of understanding IAB.

### 5.3 Qualitative Results

Our study included a qualities assessment using a questionnaire. The majority of participants commented that estimating depth perception was difficult. Several comments such as “*It was difficult to understand the depth of the tool*”, “*It was quite awkward looking at the screen and having to move my arm across here*” and “*It was tricky to know where things were in 3D and it took a few goes to know which direction you were moving in*” were made. After the second task was completed, the majority of the participants also commented on their increased ability in determining the depth of the tool on screen. This performance increase was also seen in the time decrease taken to complete the second task when compared to the first. One participant stated “*Finding the depth before we could actually extract the tumour was quite challenging. In a real-life scenario you won’t want to just move the tool around without knowing how deep it is going in the human body, until you find the organ itself*”. This highlights the importance of simulated tools like our prototype for training student surgeons, allowing them to become accustomed to the controls in relation to the level of abstraction - in this case, users needed to focus on the external screen. Future research should investigate the use of head mounted displays such as the Microsoft HoloLens for providing a direct augmented view of the operating area.

Participants who chose not to comment on depth perception interpreted the question in another way and instead commented on other parts of the experiment like the bright green colour of the tumour and the lines of the wire frame visualisation technique. Comments were made about the general perceived assistance felt by having coloured lines from the wireframe overlay condition including, “*the second time was easier compared to the first one, the lines on the screen helped me find the tumour, I found them very useful*”. Similarly comments were made about the specific colours of the overlays like “*It was easier to see the tumour this time due to the contrast between the background and the lines*” and “*the red colour was better as the tumour was marked clearly and hence it was easier to find*”. While the colour of the overlay seems like an obvious aspect to consider for these systems, there may also be a need for the system to be adaptive based on the type of surgery in order for the content to stand out.

## 6 Conclusion

In this paper we presented an evaluation of an AR laparoscopy prototype to assist surgeons through keyhole surgery tasks. We ran a within-subject study of the prototype, exploring the effects of colour, opacity and information representation of the AR overlay. The results point to the potential of these systems to guide new surgeons and students through tasks while increasing their depth perception when using a laparoscopic camera and tools. At present we have insufficient data to draw any firm conclusions, however, we have demonstrated the potential of our approach and provide a firm basis for future work. Future work should aim to evaluate keyhole surgery using other AR hardware form factors, such as head mounted displays, and test AR’s performance with actual surgeons.

## Footnotes

1
https://www.3bscientific.co.uk
